# The effect of zeolite incorporation on the physical properties of silver-reinforced glass ionomer cement

**DOI:** 10.1007/s10856-022-06659-2

**Published:** 2022-04-11

**Authors:** Spencer Lang, Jessica Hao, Francis Mante, Kresimir Pavelic, Fusun Ozer

**Affiliations:** 1 Department of Preventive and Restorative Sciences, School of Dental Medicine, University of Pennsylvania, Philadelphia, PA USA; 2 Department of Biology, College of Arts and Sciences, University of Pennsylvania, Philadelphia, PA USA; 3 Faculty of Medicine, Juraj Dobrila University of Pula, Pula, Croatia

**Keywords:** Glass ionomer cements, Zeolite clinoptilolite, Dental restorative materials, Physical properties, Biomaterials

## Abstract

Zeolite can impart antibacterial properties to dental materials in the long-term when incorporated with inorganic cations. However, due to its porosity, it may jeopardize the mechanical integrity of the dental material. The aim of this project was to determine the effect on physical properties when zeolite is added to commercially available Ag-reinforced Glass Ionomer Cement (GIC). Sample groups were prepared according to the percentage of zeolite-clinoptilolite (0% - control, 0.5%, 1%, 2%, and 4% wt) added to Ag-GIC. Water sorption, solubility, Vickers hardness, and flexural strength were determined. Specifically, 10 × 2 mm circular disks were fabricated for the Vickers hardness, water sorption, and water solubility tests and 25 × 5 × 2 mm bars were created for the flexural strength test. The results from the surface hardness, water sorption, and flexural strength tests suggested that adding 0.5–4% wt of zeolite to Ag-reinforced GIC did not diminish its physical properties. However, the water solubility results showed that higher concentrations (2–4% wt) of zeolite had a statistically significant increase in water solubility compared to the control. Up to 4% wt zeolite can be incorporated into Ag-reinforced GIC without compromising mechanical properties. Incorporation of 0.5–1% wt zeolite to Ag-reinforced GIC will maintain an adequate surface hardness, water sorption, and flexural strength without compromising water solubility. Further research is needed to determine the effects of higher water solubility on clinical efficacy of zeolite modified Ag-GIC.

**Figure:**
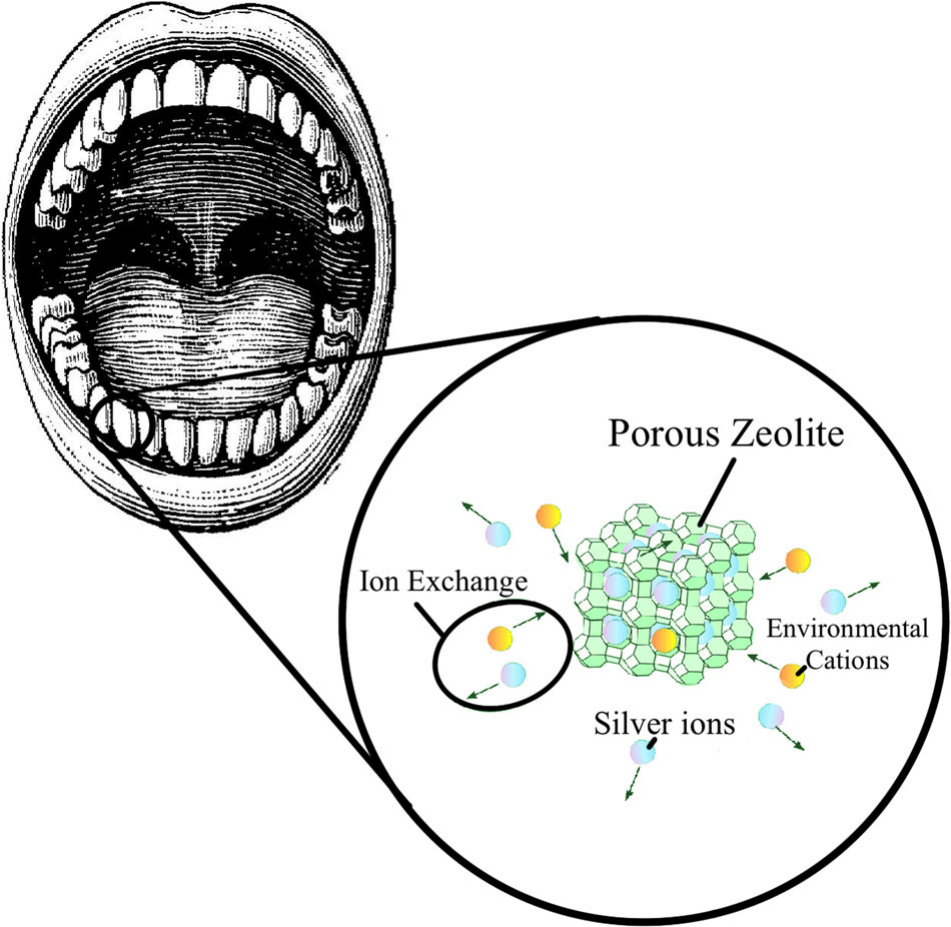
Graphical abstract

Graphical abstract

## Introduction

Glass Ionomer Cements (GIC) have been widely used in dentistry as a base and restorative material, especially in primary teeth and for Atraumatic Restorative Treatments (ART) [1]. Due to its biocompatibility and continuous fluoride-releasing properties, GIC can be used for the prevention of recurrent caries [2, 3]. However, the small amount of fluoride released by GIC only has a minimal antibacterial effect [4–6]. There is a need for a cost-effective, antibacterial material to prevent recurrent caries without affecting the physical properties and longevity of the restoration. Specifically, the effectiveness of the GIC could be improved if it was combined with an antibacterial agent to reduce the surrounding biofilm bacteria.

Zeolite is a porous natural earth mineral with a tetrahedral crystalline structure made of SiO_4_ and AlO_4_, which is very similar to the composition of GIC. Due to its porosity, zeolite can readily undergo cationic exchange with the surrounding environment [7]. Researchers have been able to harness this unique characteristic of zeolite by incorporating antibacterial cations, such as silver, into the pores. This process is aided by zeolite’s strong affinity for silver (Ag) ions and its ability to electrostatically bind to the ions for up to ~40% in its framework [8]. Zeolite can then release these antibacterial ions in a controlled manner by slowly exchanging them with environmental cations. This only takes place when moisture is present and continues until the silver concentration meets the local equilibrium value [9]. This mechanism allows for the release of silver ions over a long period of time to inhibit the growth of pathogenic bacteria. Silver ions are known to act on microbes through 3 general mechanisms: (1) perforating the bacterial cell wall through reactions with peptidoglycan [10, 11], (2) inhibiting cellular respiration and generating reactive oxygen species to disrupt metabolic pathways [10, 12], and (3) disrupting DNA replication [10, 13]. Considering the potent antimicrobial properties of silver ions and its compatibility with the low-cost zeolite, using both materials in conjunction with one another may serve as an economical way of increasing the antimicrobial effectiveness of GICs in the long term to prevent secondary caries [8, 9, 14].

When zeolite is combined into Ag-reinforced GIC to enhance its antibacterial properties, it is equally important to consider the impacts on the physical properties as well. Dental materials such as GIC must be able to withstand a variety of everyday forces, from mastication to speech, without compromising their structural integrity and strength. Some of the most common physical properties studied in dental materials include flexural strength, bond strength, compressive strength, setting time, and surface microhardness [15]. However, there has been very little research investigating the physical properties of GIC when combined with zeolite and silver ions. Despite showing promising results, the physical tests for zeolite and silver ions in GIC are not yet comprehensive [16]. To the best of our knowledge, only data on bond strength [17–19] and compressive strength [12, 19] are available. Given the importance of physical properties to dental materials, it is critical to investigate a broader variety of physical properties to adequately gauge the potential of the material to be incorporated into practice.

In this study, various concentrations of zeolite were incorporated into Ag-reinforced GIC to determine its effects on the physical properties of the material. Ag-reinforced GIC has been shown to possess silver ion release properties that may result in an antibacterial effect when used in dental restorations [20]. However, the amount of silver released into the environment is rather low, and a new method should be investigated to bolster the antibacterial effects of the material [20]. We hypothesize that the addition of zeolite will prolong the silver ion release of Ag-reinforced GIC while improving the antibacterial properties without diminishing its physical properties. Specifically, this paper reports on the physical properties (Vickers surface hardness, water sorption, water solubility, and flexural strength) of various concentrations of zeolite-clinoptilolite in Ag-reinforced GIC as observed in preliminary laboratory experiments. If there is a concentration of zeolite that has minimal detrimental effects on the physical properties of Ag-reinforced GIC, further experiments, such as antimicrobial and ion-release tests, will be conducted. These experiments will improve knowledge on all aspects of the material, including its potential towards reducing the rate of oral infections and diminishing the proclivity of dental restoration failures.

## Materials and methods

### Sample preparation for Vickers hardness, water sorption, and water solubility tests

Previous studies have generally combined zeolite at 0.2–5% when testing for antibacterial and physical properties [12, 17–19]. Therefore, to maintain comparative values, the present study tested five zeolite-clinoptilolite (Panaceo International GmbH, Carinthia, Austria) concentrations of 0%, 0.5%, 1%, 2%, and 4% wt incorporated into Ag-reinforced GIC (SDI Limited, Bayswater, Australia).

Using the standard flat scoop provided in the Riva Silver kit, three scoops of Riva Silver powder were weighed using an analytical scale with an accuracy of 0.0001 g. An aliquot of the measured powder equivalent to the intended percent by weight of zeolite was removed, and zeolite was added until the original weight was achieved. The powders were transferred to a mixing pad, and gently mixed by hand with a metal spatula until homogenous. Following the instructions from the manufacturer, three drops of the Riva silver liquid was added to the powders to obtain an approximate 7:1 powder-liquid ratio. The liquid and powder were mixed by hand with a metal spatula for 30 s until homogenous throughout.

After mixing, the cement was immediately placed in excess into a premade plastic mold that formed 10 mm × 2 mm circular disks. A thin layer of petroleum jelly was first applied on the internal surfaces of the plastic mold to facilitate extraction of the disks. After adding the material, the mold was placed between glass slabs and held down by a 250–300 g weight to ensure the formation of smooth surfaces on the disks. Following the instructions of the manufacturer, the samples were allowed to sit for at least 6 min until the material set. After extracting the disks from the mold, extruded excess was removed using a scalpel. This procedure was repeated so that 12 samples per group were made for the water solubility/sorption tests, and 10 samples per group were made for the Vickers hardness test. The Vickers hardness test disks were wrapped in wet gauze in a sealed container and placed in an incubator at 37 °C for 24 h until use to mimic the conditions of the oral cavity. The water sorption/solubility samples were stored in a dry, empty petri dish until use.

### Vickers hardness test

Two sets of Vickers hardness measurements were taken for the samples via a Leco Microhardness Tester machine (Model type M-400-G1, LECO Corporation, St. Joseph, USA). The first set of measurements were taken prior to the water sorption test, and the second set was taken 1 week into the water sorption test to compare the change in surface hardness after storage in water for one week.

Three indentations were made for each of the 10 samples in every test group with a load of 500 g for 10 s dwell time. The indentation lengths were measured immediately after indentations to minimize the possibility of viscoelastic recovery. The Vickers hardness was automatically calculated by the machine and averaged amongst the three values and 10 samples.

### Water sorption and water solubility tests

The water sorption test utilized 12 samples of 10 mm × 2 mm circular disks from each of the five zeolite concentration groups. Using a digital caliper, the diameter and height of each sample were measured to an accuracy of 0.01 mm. The volume of each sample was then calculated using the following formula:

$${{{\mathrm{V}}}} = \pi \left( {{{{\mathrm{d}}}}/2} \right)^2{{{\mathrm{h}}}} = \pi {{{\mathrm{r}}}}^2{{{\mathrm{h}}}}$$

d = measured sample diameter (mm); h = measured sample height (mm); r = calculated sample radius (mm); V = calculated sample volume (mm).

The initial mass (M_1_) of each sample was determined on an analytical scale with an accuracy of 0.0001 g. Each sample was placed into a separate well in a 12-well plate and submerged in a deionized water bath to mimic the presence of saliva in the oral cavity. The plates were incubated at a constant 37 °C, which mimicked the temperature of the human body. Every 24 h, the samples were blotted dry completely until there were no visible water droplets on the sample, and their masses were measured on the analytical scale.

After a constant mass (M_2_) was reached for each group, the samples were placed in a desiccator containing anhydrous calcium sulfate and cobalt chloride (Thermo Fisher Scientific, Waltham, MA, USA) at room temperature. The calcium sulfate served as a desiccant and the cobalt chloride acted as a visual indicator, which transitioned from blue to pink upon absorbing the water from the Ag-reinforced GIC samples and surrounding air in the desiccator. After every 24 h in the desiccator the samples were weighed on the analytical scale, weighing was repeated daily until a constant mass was achieved (M_3_).

Water sorption (W_sp_) and water solubility (W_sl_) were calculated using the constant masses determined above using the following formulas [21]:

$$\begin{array}{l}{{{\mathrm{W}}}}_{{{{\mathrm{sp}}}}} = \left( {{{{\mathrm{M}}}}_2 - {{{\mathrm{M}}}}_3} \right)/{{{\mathrm{V}}}}\\ {{{\mathrm{W}}}}_{{{{\mathrm{sl}}}}} = \left( {{{{\mathrm{M}}}}_1 - {{{\mathrm{M}}}}_3} \right)/{{{\mathrm{V}}}}\end{array}$$

M_1_ = conditioned mass prior to water immersion (g); M_2_ = sample mass after water immersion (g); M_3_ = reconditioned sample mass after drying (g)V = calculated sample volume (mm).

### Flexural test

The flexural strength (F_s_) of the zeolite-incorporated Ag-reinforced GIC samples were evaluated according to the specifications outlined in the ISO Standard ISO 9917-2 and Bonifacio et al. [22, 23]. Bar-shaped 25 × 5 × 2 mm molds were prepared using Aquasil EasyMix Putty (Dentsply Sirona, York, PA, USA). The Ag-reinforced GIC samples were prepared and mixed according to the manufacturer’s instructions and placed in the molds for 6 min to set. After removing the samples from the mold, excess material was carefully trimmed using a scalpel, and the height and width of the specimens were measured using a digital caliper to an accuracy of 0.01 mm. Ten sample bars that were devoid of porosity were selected for flexural testing. This process was repeated for each concentration of zeolite (0.5%, 1%, 2%, and 4%) so that there were 10 bars in each of the four concentration groups. After fabrication, the samples were placed in 100% humidity and incubated at a constant 37 °C for 48 h until testing.

Using a universal testing machine (Instron no. 4204, Instron Corp, Canton, MA, USA), the samples were subjected to a 3-point bending test with 16.65 mm between the supports. A loading force was applied at a constant crosshead speed of 1 mm/min until the sample bar fractured. The load at fracture was used to determine the flexural strength (scale factor for load = 0.5 kN/V). The flexural strength (σ, in MPa) was calculated from the equation [24]:

$$\begin{array}{l} {{{{\mathrm{F}}}} = {{{\mathrm{maximum}}}}\,{{{\mathrm{load}}}}\,{{{\mathrm{exerted}}}}\,{{{\mathrm{on}}}}\,{{{\mathrm{the}}}}\,{{{\mathrm{specimen}}}}\left( {{{\mathrm{N}}}} \right)} \\ {{{{\mathrm{l}}}} = {{{\mathrm{the}}}}\,{{{\mathrm{distance}}}}\,{{{\mathrm{between}}}}\,{{{\mathrm{the}}}}\,{{{\mathrm{supports}}}}\,\left( {16.65\,{{{\mathrm{mm}}}}} \right)} \\ {{{{\mathrm{b}}}} = {{{\mathrm{the}}}}\,{{{\mathrm{specimen}}}}\,{{{\mathrm{width}}}}\,\left( {{{{\mathrm{mm}}}}} \right)}\\ {{{\mathrm{h}}}} = {{{\mathrm{specimen}}}}\,{{{\mathrm{height}}}}\left( {{{{\mathrm{mm}}}}} \right).\end{array}$$

### Statistical analysis

The Vickers hardness data sets were further analyzed via *t* test (α = 0.05) to test for differences in the surface integrity of the sample groups and the effect of time. The mean values of water sorption, water solubility and flexural strength properties were evaluated using a Kruskal–Wallis test at α = 0.05. Initially one-way ANOVA tests were attempted. However, the equal variance and normality tests were not always passed for the data. Therefore, the authors decided to use the aforementioned tests in place of the ANOVA.

## Results

### Surface hardness or Vickers test

**Fig. 1:**
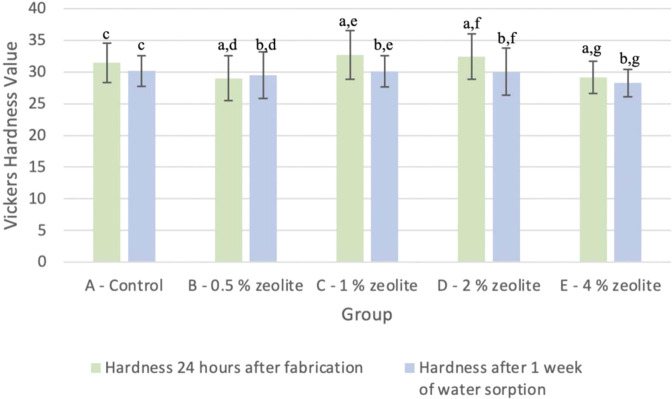
Mean Vickers surface hardness of various concentrations of zeolite in Ag-reinforced GIC before and after 1 week of storage in water (*n* = 10). Note: Means with letters a/b did not have a statistically significant difference (*p* > 0.05) when compared to their respective controls. All means 24 h after fabrication did not have a statistically significant difference (*p* > 0.05) compared to means 1 week after water sorption. This is shown by letters c–g across the concentration groups

Figure 1 shows the surface hardness values for the five test groups (control - 0%, 0.5%, 1%, 2%, 4%) comparing data 24 h after fabrication and after one week of water sorption tests. Using a *t* test at α = 0.05, the difference between the means at each time was compared for all five test groups. When comparing the mean hardness values 24 h after fabrication and after one week of water sorption for any of the concentration groups for the control (*P* = 0.293), 0.5% (*P* = 0.778), 1% (*P* = 0.086), 2% (*P* = 0.153), and 4% (*P* = 0.419), there was no significant difference. Next, *t* test at α = 0.05 was used to compare the difference between control vs. 0.5%, 1%, 2%, and 4% at each time. There was no statistically significant difference between the control and the 0.5% (*P* = 0.105), 1% (*P* = 0.409), 2% (*P* = 0.492), and 4% (*P* = 0.072) groups at 24 h after fabrication. In addition, there was no statistically significant difference between the control and the 0.5% (*P* = 0.615), 1% (*P* = 969), 2% (*P* = 0.923), and 4% (*P* = 0.078) groups after one week of water sorption.

### Water sorption and water solubility tests

**Fig. 2:**
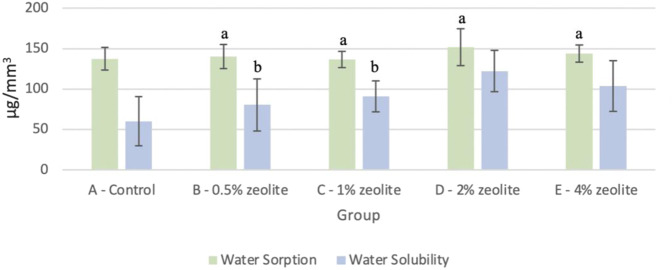
Mean water sorption and water solubility of various concentrations of zeolite in Ag-reinforced GIC (*n* = 12). Note: The letter a represents the absence of a statistically significant difference between the mean of the sample group as compared to the water sorption control (*p* > 0.05). The letter b indicates that there is no statistically significant difference between the mean of the sample groups as compared to the water solubility control (*p* > 0.05)

Figure 2 depicts the water sorption and solubility values calculated with the five concentrations of zeolite (0%, 0.5%, 1%, 2%, and 4%) in Ag-reinforced GIC. The mean water sorption values, which utilized the difference between sample mass after water immersion and the reconditioned mass after drying, were consistent across the concentration groups. In other words, no statistically significant difference (*p* = 0.302) was found when each experimental group was compared with the control in the water sorption tests. However, the mean water solubility values, which utilized the difference between sample mass prior to immersion and the reconditioned mass after drying, increased as the concentration of zeolite increased. Compared with the control group, the difference in the mean values for 2% zeolite (*P* = 2.37 × 10^−6^) and 4% zeolite (*P* = 3.98 × 10^−4^) were greater than would be expected by chance, so a statistically significant difference was observed. On the other hand, there is no significant difference with 0.5% (*P* = 8.47 × 10^−2^) and 1% (*P* = 9.88 × 10^−3^) zeolite compared to the control. The lowest water solubility value (60.21 ± 30.26 μg/mm^3^) was for the control group (0% zeolite) with increasing values to the highest (122.33 ± 25.56 μg/mm^3^) for 2% zeolite.

### Flexural test

**Fig. 3:**
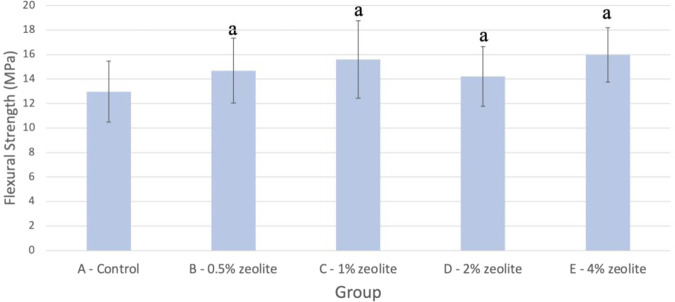
Mean flexural strength (F_s_ in MPa) of various concentrations of zeolite in Ag-reinforced GIC (*n* = 10). Note: Means with the letter a did not have a statistically significant difference when compared to the control

Figure 3 shows the flexural strength (MPa) calculated for the groups with standard deviation and significance values. Using a Kruskal–Wallis test at α = 0.05, the difference between the means at each time was compared for all five test groups. It was determined that there wasn’t a statistically significant difference between the mean values of the control and 0.5% (*P* = 0.151), 1% (*P* = 0.052), 2% (*P* = 0.274), as well as 4% (*P* = 0.0712).

## Discussion

### Surface hardness or Vickers test

Vickers hardness is one of the most common physical properties tested to ensure the durability of dental materials in withstanding everyday forces and wear. Specifically, the surface of the material must be able to resist deformation caused by indentation, penetration, scratching, and abrasion [25]. In the oral environment, dental materials are exposed to a plethora of surface-eroding factors such as saliva, microorganisms, tooth brushing, food substances, and mastication [26]. Consequently, it is crucial to consider the impact on the surface hardness when adding zeolite to Ag-reinforced GIC to ensure their optimal long-term clinical performance. Although the results from the current study suggest that zeolite at 1–2% by weight had the highest surface hardness, none of the experimental groups had a significant difference when compared with the control. Therefore, zeolite at any concentration up to 4% wt does not hinder the microhardness of Ag-reinforced GIC.

### Water sorption and water solubility tests

There are many factors, such as the presence of moisture, within the oral cavity that may influence the properties of restorative materials. To simulate in vivo conditions, the samples were stored in a 37 °C-water bath (water sorption test) followed by a desiccator (water solubility test). Water sorption and water solubility are commonly tested properties since erosion due to varying levels of moisture may directly affect the degradation of the Ag-reinforced GIC. Specifically, water sorption may change the physical properties of GICs through lamination and degradation, while solubilization may cause a loss of material mass, decrease in mechanical properties, and increase in rate of restoration failure [21, 27, 28]. Thus, it is important to understand the storage solution’s influence on different concentrations of zeolite-incorporated Ag-reinforced GIC over a period to predict the behavior of the restorative material in the long term.

The Ag-incorporated GIC water sorption and solubility data obtained in this present study were consistent with the previously published values. Specifically, water sorption of GIC ranged from 81.60 to 143.76 μg/mm^3^ after 7 days in deionized water, and the solubility values ranged from 36.76 to 115.21 μg/mm^3^ [21]. Based on the results from our study, by incorporating increasing amounts of zeolite into Ag-reinforced GIC, the results of the water sorption test were not statistically different from the control. However, as increasing amounts of zeolite was added, the results of the water solubility test increased drastically compared to the control. This suggests that hydrating and dehydrating the Ag-reinforced samples may directly affect the structural integrity of the sample, and lower concentrations of zeolite may be more favored as it has more similar water solubility values as the control. This should be a topic of further investigation through increasing the sample size and testing groups with higher concentrations of zeolite.

### Flexural test

Despite the prevalence of GIC usage in dentistry, one of the major drawbacks of the material is its relatively low fracture strength and higher occlusal wear rate compared to modern resin composite materials and amalgam [29]. In a literature review, Hickel and Manhart showed that GIC has an annual failure rate of around 1.4–14%, with bulk fracture being the primary reason for failure in posterior teeth [30]. Since poor fracture strength of the material can contribute to higher long-term restoration failure rates, flexural strength should be among the physical properties tested to ensure that zeolite-incorporated Ag-reinforced GIC is wear resistant enough to withstand everyday forces without compromising its strength.

The flexural test data obtained in this present study were similar to the values published from previous studies, which generally ranged from 14.2–23.1 MPa in a similar time range [22, 28]. Through a Kruskal–Wallis test, it was determined that there was no statistical significance in any of the test groups compared to the control. Therefore, zeolite at any concentration up to 4% wt does not hinder the flexural strength of Ag-reinforced GIC.

### Natural vs. synthetic zeolite

While most published studies observed the uses of synthetic zeolite, the current study incorporated natural zeolite-clinoptilolite to Ag-reinforced GIC for a variety of reasons. First, natural zeolites have a high affinity towards heavy metal ions in comparison to synthetic zeolites [31–33]. This property likely facilitates the uptake of silver ions by the zeolite when mixed with the Ag-reinforced GIC. In addition, the channel diameters of natural zeolite are very small (for example, clinoptilolite has a diameter of 0.30–4 nm), which enhances its selectivity by inhibiting the absorption of organic compounds and large gas molecules [34]. Furthermore, synthetic zeolites are more expensive than their natural counterparts due to the need for appropriate equipment, clean substrates, and energy [34]. If zeolite-incorporated Ag-reinforced GIC was to be commonly used and widely distributed in dental procedures, then the more cost-efficient option should be explored.

## Conclusion

The results from our surface hardness, water sorption, and flexural strength tests suggest that adding any concentration, from 0.5–4% of zeolite by weight, to Ag-reinforced GIC does not diminish its physical properties. However, the water solubility results showed that higher concentrations (2–4% wt) of zeolite had a statistically significant increase in water solubility compared to the control. Further research should be conducted to determine the extent of the effects that higher water solubility values may have on the physical integrity of the material.

Although none of the statistical analyses showed significant changes in Vickers hardness, water solubility, and flexural strength as a result of zeolite percentage, the authors recommend adding 0.5–1% wt zeolite to Ag-incorporated GIC. This is because 0.5–1% wt zeolite showed the least deviation from the control in water solubility while increasing in flexural strength. These results are used in addition with previous literature, which has shown that zeolite improves the antibacterial properties of Ag-GIC from as low as 0.2% [8, 19]. However, before a definitive recommendation can be made about the optimum zeolite composition, further tests on silver ion release and antibacterial effectiveness must be completed. Specifically, antibacterial tests should be conducted in the future to determine the effectiveness of zeolite and Ag-reinforced GIC towards hindering the growth of a variety of oral microorganisms. Future studies will investigate the silver ion release profile of zeolite-incorporated Ag-reinforced GIC to determine the longevity and quantity of silver ion release. If all the tests continue to show an unaffected mechanical profile in combination with antimicrobial effectiveness, using zeolite in low concentrations will prolong the time period that the antibacterial ions are released from Ag-reinforced GIC. This creates the potential for a cost-effective solution to preventing recurrent caries while maintaining long-term restoration quality.
